# Comparison of Two Quantitative Methods of Discerning Airspace Enlargement in Smoke-Exposed Mice

**DOI:** 10.1371/journal.pone.0006670

**Published:** 2009-08-18

**Authors:** Richard E. Jacob, James P. Carson, Kathy M. Gideon, Brett G. Amidan, Cathie L. Smith, K. Monica Lee

**Affiliations:** 1 Biological Monitoring and Modeling, Pacific Northwest National Laboratory, Richland, Washington, United States of America; 2 Pathology and Toxicology, Battelle Toxicology Northwest, Richland, Washington, United States of America; 3 Statistics and Sensor Analytics, Pacific Northwest National Laboratory, Richland, Washington, United States of America; 4 Health and Life Sciences, Battelle Memorial Institute, Columbus, Ohio, United States of America; Emory University, United States of America

## Abstract

In this work, we compare two methods for evaluating and quantifying pulmonary airspace enlargement in a mouse model of chronic cigarette smoke exposure. Standard stereological sample preparation, sectioning, and imaging of mouse lung tissues were performed for semi-automated acquisition of mean linear intercept (L_m_) data. After completion of the L_m_ measurements, D_2_, a metric of airspace enlargement, was measured in a blinded manner on the same lung images using a fully automated technique developed in-house. An analysis of variance (ANOVA) shows that although L_m_ was able to separate the smoke-exposed and control groups with statistical significance (p = 0.034), D_2_ was better able to differentiate the groups (p<0.001) and did so without any overlap between the control and smoke-exposed individual animal data. In addition, the fully automated implementation of D_2_ represented a time savings of at least 24x over semi-automated L_m_ measurements. Although D_2_ does not provide 3D stereological metrics of airspace dimensions as L_m_ does, results show that it has higher sensitivity and specificity for detecting the subtle airspace enlargement one would expect to find in mild or early stage emphysema. Therefore, D_2_ may serve as a more accurate screening measure for detecting early lung disease than L_m_.

## Introduction

The development and use of animal chronic obstructive pulmonary disease (COPD) models requires sensitive methods of monitoring and quantifying the disease progression. Key components of COPD, as defined by the American Thoracic Society, are “abnormal, permanent enlargement of airspaces distal to terminal bronchioles, accompanied by destruction of their walls” [1]. In addition, destruction in emphysema, a major component of COPD, is defined as “nonuniformity in the pattern of respiratory airspace enlargement” [2]. In mild emphysema, it has been shown that increases in lung volume are not necessarily accompanied by decreases in total surface area [3]. The increase in volume may be due to the deterioration of elastic fibers in parenchymal tissue, which can lead to breakage of weakened alveolar walls that are under mechanical stress [4]. Although this breakage may result in a slight loss of total surface area, it will likely lead to a few enlarged airspaces that are surrounded by smaller, intact ones.

The mean linear intercept (L_m_), a measure of the surface area to volume ratio, is by and large the most commonly reported metric of emphysema. However, its application and interpretation tend to vary among different laboratories, and results are often misused as an assessment of airspace diameter or airspace size [5]–[7]. In cases of mild emphysema, in which diseased areas of the lung may be small, dispersed, and heterogeneous with respect to distribution of airspace sizes (e.g. see Refs. [8]–[10]), it is generally difficult to quantify disease severity, as conventional methods, such as L_m_, employ numerical averaging to extract a “central tendency” [6] and, hence, tend to underestimate the important influence of subtle localized changes or outliers. This was pointed out in Ref. [7]: “L_m_ is much more difficult to measure and fraught with danger of bias if the airspace size is very variable.” There are compelling arguments against abandoning L_m_ [11], although these views highlight that L_m_ may not be the most sensitive indicator for early emphysema diagnosis. Indeed, several studies have demonstrated that L_m_ often cannot distinguish mild emphysema from healthy controls [12]–[17]. Therefore, a histological method of measuring airspace enlargement that is specifically sensitive to the presence of the largest airspaces is desirable for detecting such a disease state.

Recently, Parameswaran et al. [18] introduced non-conventional metrics that could potentially be used as indicators of heterogeneously distributed airspace sizes characteristic of early lung disease. Briefly, these indexes, referred to as D_1_ and D_2_ (described in detail and derived in Ref. [18]), utilize the equivalent airspace diameters (i.e. diameter of a circle of equivalent area) and then incorporate higher moment factors from the airspace diameter distributions. Thus, the largest airspaces—potential indicators of early disease state—are weighted more heavily than smaller ones. We stress that D_1_ and D_2_ do not provide conventional 3D stereological information about average airspace dimensions – they simply emphasize the presence of a minority of enlarged airspaces. Nevertheless, as observed in Ref. [19], these new indexes may prove useful as indicators of physiology expected in early or mild emphysema but require rigorous validation.

Herein, as a validation effort, we have applied these indexes post factum to a study of airspace enlargement in smoke-exposed mice and compared the results to conventional L_m_ measurements on the same histological images.

## Methods

### Ethics Statement

Smoke exposure took place at Washington University in St. Louis. Experimental procedures were approved by the Institutional Animal Care and Use Committee of Washington University in St. Louis. Animals were allowed access to food and water ad libitum and were humanely sacrificed as necessary to ameliorate suffering.

### Lung Sample Preparation

**Figure 1:**
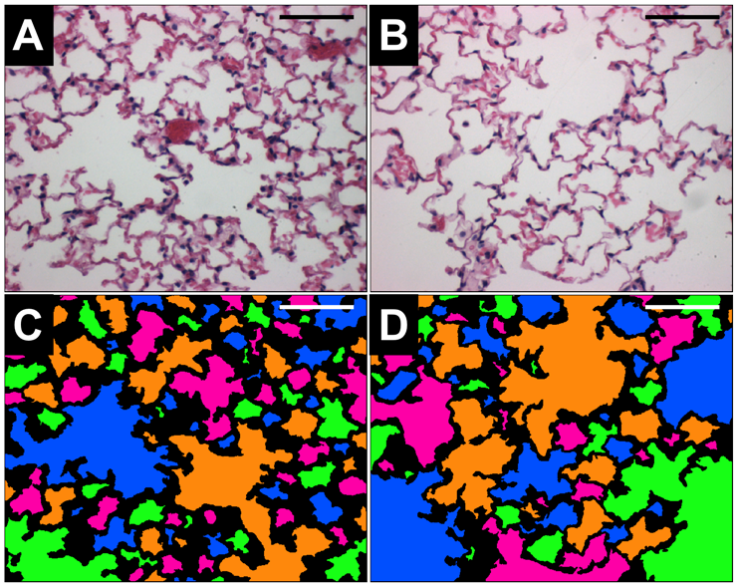
Representative H&E stained images from a control mouse (A) and a smoke-exposed mouse (B). Color maps of each image, (C) and (D) respectively, are shown to illustrate the different airspaces. The bars are 200 µm.

Lung tissue samples from 20 female AKR/J mice were used in this study, with 10 exposed to mainstream cigarette smoke (2–4 cigarettes/day, 6 days/week) for 24 weeks and 10 age-matched controls, as described previously [20], [21]. At the end of smoke exposure, the mice were sacrificed by CO_2_ asphyxiation and exsanguinated (the vasculature was not flushed with saline). Next, the chest cavity was opened and the diaphragm incised. Lungs were then inflated to 25 cmH_2_O with 10% neutral buffered formalin for ≈10 minutes, after which the trachea was tied off and the lungs excised and placed in a formalin bath for≥2 days. After fixation, lungs were trimmed and randomly oriented in preparation for sectioning. Lungs were embedded with paraffin and sectioned into 5 µm thick slices that were stained with hematoxylin and eosin (H&E). Slices were made in random directions, and eight random slices selected from all lobes of each mouse were placed on a slide. We note that this random method will result in some lobes being sampled multiple times, and the possibility exists that some lobes will avoid sampling altogether. Slices were then imaged at 200×magnification using a Nikon Optishot II microscope and Zeiss Axiocam digital camera; 12 images per mouse were acquired. Image locations were selected by using a random number generator (www.random.org) to determine image coordinates. Major airways and vasculature were generally avoided in selecting fields to focus on peripheral parenchyma, as reported by others (cf. Refs. [22]–[25]). When one of these was encountered, the microscope field was shifted in a randomly selected direction until the field included parenchymal tissue only. Digital images were 606×480 pixels and covered a field of approximately 1.0 mm×0.8 mm. Figure 1 shows representative H&E stained images from control (A) and smoke-exposed (B) mice, with color maps included to aid the eye in distinguishing airspaces (C and D, respectively). We note that gross examinations of morphometry of all healthy vs. smoke-exposed mice were insufficient for definitively determining the severity of disease.

### Mean Linear Intercept Measurements

**Figure 2:**
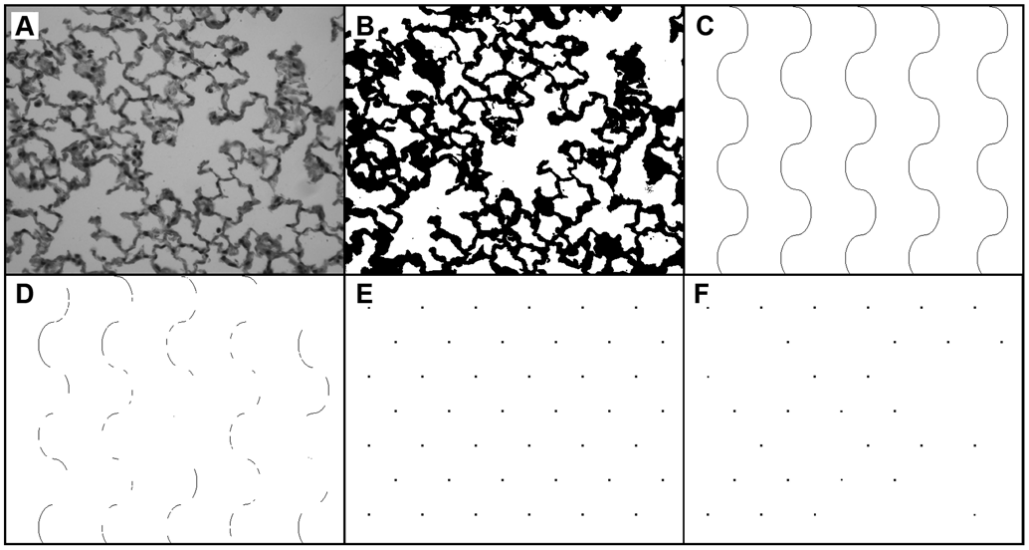
Image analysis steps for calculating L_m_. A) H&E stained image acquired at 200×magnification. B) Threshold of image distinguishes tissue from airspace. C) Cycloid grid lines. D) Intersection of cycloid lines with tissue in thresholded image (B). E) Grid points. F) Intersection of grid points with airspace in thresholded image.

The mean linear intercept (L_m_) was measured on the lung section images using Image-Pro® Plus (Media Cybernetics, Bethesda, MD) image analysis software as described previously [26]; see Figure 2. Briefly, a binary threshold mask of the alveolar septa was made, a grid of 5 cycloid lines was placed on the mask [10], [27], and the intercepts with the septa were counted. Next, a similar mask of the alveolar airspaces was made, a grid of 42 points was placed on the mask, and the points overlaying the airspaces were counted; truncation of airspaces by the optical boundary was ignored. Counts of non-alveolar airspace or tissues were manually removed and L_m_ was then calculated using the following equation:
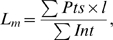
(1)where Σ*Pts* is the sum of the points in the airspace mask, *l* is the cycloid length per point (including a geometrical correction for the curvature of the cycloid lines), and Σ*Int* is the sum of the intercepts of the cycloid lines with alveolar septa. The semi-automated L_m_ measurements required ≈2–3 minutes per image for complete analysis.

### D_2_ Measurements

Automated measurements of D_2_ were performed on the same images after the completion of the L_m_ measurements. To eliminate potential bias, neither the L_m_ data nor the exposure histories of the mice were available a priori to individuals calculating D_2_.

The indexes D_1_ and D_2_ are derived from the ratios of the distributions of the standardized moments of the mean equivalent airspace diameter (i.e. the diameter of a circle of equivalent area: 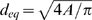). Hence, D_1_ is defined as the ratio of the second moment to the first moment, and D_2_ is the ratio of the third moment to the second moment [18]. The calculation of these indexes requires measurement of the areas of the individual airspaces and the calculation of the equivalent diameter *d_eq_* of each airspace. Then the mean (D_0_), the variance (σ^2^), and the skewness (γ) of the *d_eq_* distribution are used to calculate D_1_ and D_2_, according to [18]:
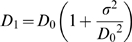
(2)

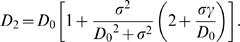
(3)


Because D_2_ includes information about both the variance and skewness of the distribution, it is expected to be more sensitive than D_1_ or D_0_ to the presence of outliers. We note that higher order indexes D*_n_* would include the (*n*+1)^th^ standardized moment of the distribution, but their implementation may be unnecessary, as D_2_ may be sufficiently sensitive, or impractical, since D*_n_* becomes increasingly complex with increasing *n*.

**Figure 3:**
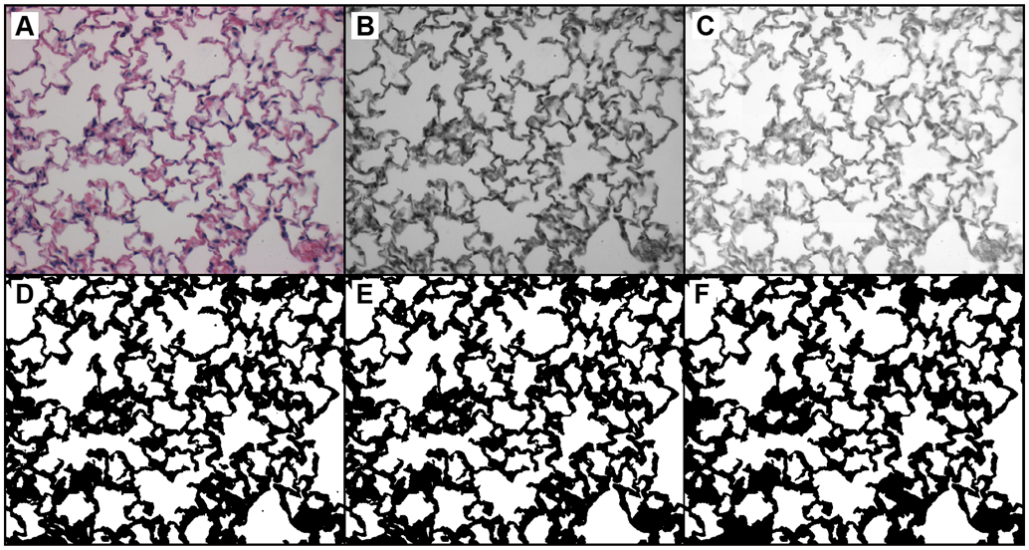
Automated steps for processing H&E stained images prior to calculating D_2_. A) RGB color image acquired at 200×magnification. B) Green-channel of A. C) Localized background intensity normalization performed on B. D) Threshold of intensity converts C to a binary image. E) Small black particles removed from D. F) Small white particles removed from E.

The following automated steps were used for calculating D_2_ from color RGB images of lung samples (see Figure 3). First, each 24-bit RGB image was converted to an 8-bit grayscale image by extracting the green channel, which provides the greatest contrast between the background and the red-blue H&E stained tissue. Then, a localized background normalization was performed to remove the differences in light intensity across each image [28]. This step linearly shifted the intensities of pixels in 30 local regions so that the maximum pixel value in each local region would be set to 255 (i.e., white). Next, each 8-bit grayscale image was converted to a binary image using a threshold of 225, with pixel values above 225 indicating airspace, and pixels at or below 225 indicating tissue (this level was set empirically). After thresholding, stray particles, or unconnected groups of edge-adjacent pixels, of area≤500 pixels were erased. Similarly, small white particles of area≤100 pixels within tissue walls were filled in. The remaining white regions represented the airspaces for the D_2_ calculation. Finally, the number of pixels in each region was measured as the area of each airspace. For each mouse, the airspace areas from all 12 images were assembled into a single data set. D_2_ was then calculated for each mouse using Eq. [3]. This automated procedure was implemented using the python programming language (www.python.org) and the python imaging library (www.pythonware.com). The fully automated D_2_ measurements required ≈20 minutes to process all 240 images, or ≈5 seconds per image, using a 3.2 MHz Pentium 4 desktop PC with 3 GB of RAM.

D_2_ was measured manually (i.e. with little or no automation) on a subset of the images to validate that the automated thresholding method did not misinterpret features. Images were chosen using a random number generator to select one image per animal; thus, 20 images were analyzed manually. As with the automated D_2_ measurements, the manual measurements were performed blind with no knowledge of treatment history. Moreover, the computer-generated threshold images were not made available until after the completion of the manual analysis to prevent bias. All manual image processing was done using ImageJ [29] as previously described [30]. Images were first filtered with a 1.0 pixel radius Gaussian filter to eliminate speckle, then a 100 pixel radius rolling-ball background subtract filter was applied to minimize intensity variations. Next, images were thresholded, and unconnected particles were erased. Images were then manually repaired by filling in regions that did not threshold properly; this was done by directly comparing the thresholded image to the original image. Finally, the areas of the individual airways were measured and copied to a spreadsheet program for analysis. Regions with an area<50 pixels were not included in the analysis, as they generally resulted from incomplete thresholding or repair. The equivalent diameter of each airspace was calculated, and D_0_, σ^2^, and γ were then determined for each image from which D_1_ and D_2_ were then calculated. This manual technique required about 5–7 minutes per image, the bulk of which was used for the image repair (i.e. particle removal and correcting poorly thresholded regions).

We note that airspaces truncated by the borders of the image frame were included in the D_2_ analyses (both manual and automated). This was necessitated by the fact that L_m_ was calculated on the entire image frame (as is standard practice), and to make a fair comparison of L_m_ and D_2_ they must be calculated on the same exact images. We point out that the truncation may result in D_2_ measurements that are skewed to somewhat low values. However, the exclusion of these airspaces altogether only serves to filter out the largest airspaces – since they are most likely to border the edge – and thus further skew the results to even lower values. To verify this we eliminated the edge-bordering airspaces and reanalyzed D_2_ and L_m_ on all the images (data not shown). We found that although D_2_ dropped considerably for the smoke-exposed group, it was still significantly higher than for the control group (p-value<0.05). L_m_ for the smoke-exposed group, on the other hand, dropped so much that it became dramatically *lower* than for the control group (p-value<0.0005). Therefore, elimination of truncated airspaces clearly misrepresents the true nature of the lung tissue much worse than including the truncated airspaces. Hence, airspaces were defined by the edge of the optical image. Ideally, acquisition of larger image fields would be desirable so that truncated airspaces could be excluded without affecting results, as was done in Ref. [30]. This was not possible herein, as D_2_ was calculated after the completion of the L_m_ study.

### Statistical Analysis

A statistical comparison was made between manual and automatic measurements of D_0_, D_1_, and D_2_ to determine how well the individual measurements for the two methods correlated and whether or not there was an overall difference in mean values for each variable. Pearson correlation coefficients were calculated, and analysis of variance (ANOVA) along with two sample t-tests were used to establish if differences existed between the measurement methods. ANOVA procedures were also performed to determine if D_2_ and L_m_ were equally effective in detecting significant differences between smoke-exposed mice and those in the control group. In these statistical analyses, a significance level (α) of 0.05 was used. Additionally, linear discriminate analysis was used to create classification rules to predict the specificity (i.e. ability to discern true negatives) and the sensitivity (i.e. ability to discern true positives) for D_2_ and L_m_.

## Results

**Figure 4:**
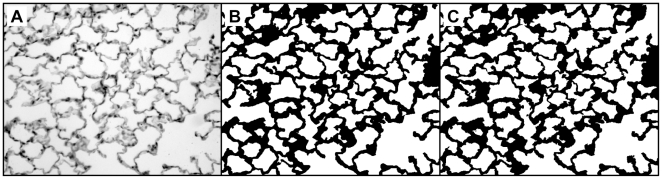
Comparison of thresholded images for manual and automated D_2_ analysis. A) 606×480 pixel image of a lung slide acquired at 200×magnification (original images were acquired in RGB color). B) Manually thresholded and repaired image. C) Automatically thresholded image. Subtle differences between the thresholding methods can be seen upon inspection.

Figure 4 shows an example of a typical lung slide image (panel A) together with the results of manual thresholding (panel B) and automatic thresholding (panel C) used for D_2_ measurements. This image demonstrates that the automated method did not introduce artifacts or misinterpret features.

**Figure 5:**
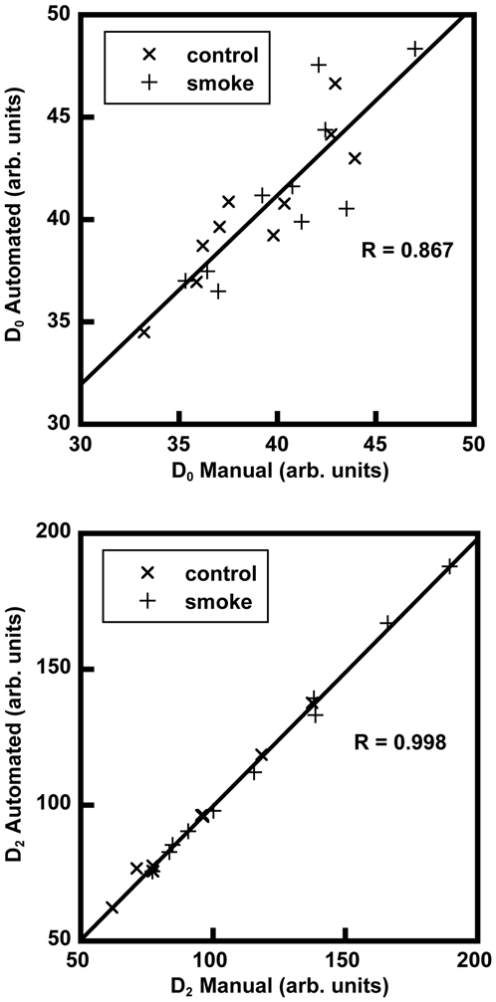
Automated vs. manual calculation of the mean equivalent diameter D_0_ (top) and weighted index D_2_ (bottom) from 20 randomly selected images. In spite of the subtle differences between thresholding methods (see Figure 4), the strong correlations indicate that there are no statistically significant differences between measurement techniques.

Graphs comparing the automatic vs. manual measurements of D_0_ and D_2_ for the 20 random images are shown in Figure 5. Results of the statistical analysis indicate that the two measurement types were highly correlated for each variable with Pearson correlation coefficients of R = 0.867 for D_0_, R = 0.994 for D_1_ (data not shown), and R = 0.998 for D_2_. The ANOVA showed that there was a significant interaction between measurement type (manual vs. automatic) and variable (D_0_, D_1_, or D_2_), indicating that the manual and automatic measurement values were inconsistently different across the variables (p-value<0.001). Two sample paired t-tests further explored this by showing that no significant differences existed between the measurement types for D_1_ (p-value = 0.652) and D_2_ (p-value = 0.374), but did show a significant difference between measurement types for D_0_ (p-value = 0.0108). This difference is shown in Figure 5, where D_0_ values are generally higher for the automatic measurements versus the manual measurements.

Table 1 shows the data used to calculate D_2_, in addition to the L_m_ results, for the 20 mice in the study. The mice are alphanumerically labeled according to control (C) or smoke-exposed (S). The average of the standard deviations of the treated mice is significantly greater than that of the control mice (p-value<0.001). This is an indicator of increased heterogeneity of airspace sizes in the treated mice.

**Table 1:** Data from the 20 mice in this study.

Mouse	N	D_0_ (µm)	σ (µm)	γ	D_1_ (µm)	D_2_ (µm)	L_m_ (µm)
C1	989	31.844	24.72	2.69	51.027	80.756	45.7
C2	1058	32.111	24.83	2.68	51.309	81.087	46.5
C3	895	34.483	25.72	2.23	53.674	79.619	59.5
C4	944	33.959	25.24	2.42	52.713	79.877	46.5
C5	1048	32.966	24.94	2.79	51.834	82.258	47.5
C6	1092	32.498	23.20	2.31	49.058	72.557	44.9
C7	981	32.006	23.98	2.66	49.986	77.964	50.1
C8	1077	30.690	22.01	2.55	46.475	70.556	45.7
C9	1043	32.877	24.90	2.74	51.737	81.757	52.8
C10	1025	31.489	23.86	2.80	49.558	78.828	47.0
Mean (SD)	1015 (62)	32.5 (1.1)			50.7 (2.1)	78.5 (3.9)	48.6 (4.5)
S1	770	37.453	32.39	3.02	65.456	111.35	57.7
S2	722	37.445	33.31	2.73	67.078	110.69	59.6
S3	896	32.934	27.25	2.92	55.481	91.998	53.5
S4	827	35.000	31.76	2.84	63.809	107.36	53.6
S5	1015	32.014	25.07	3.09	51.648	85.841	44.7
S6	944	32.151	28.48	3.44	57.378	103.46	49.8
S7	931	34.653	31.82	3.25	63.874	113.63	51.3
S8	753	35.040	35.90	3.52	71.815	135.63	59.9
S9	822	35.056	30.30	2.75	61.243	100.60	54.4
S10	846	34.443	29.40	3.08	59.532	101.67	49.4
Mean (SD)	853 (93)	34.6 (1.9)			61.73 (5.9)	106.2 (13.5)	53.4 (4.8)

C mice were control; S mice were smoke-exposed; N, total number of airspaces after thresholding; D_0_, mean equivalent airspace diameter; σ, standard deviation of the airspace distribution; γ, skewness of the airspace distribution; D_1_ and D_2_, weighted indexes; L_m_, mean linear intercept.

**Figure 6:**
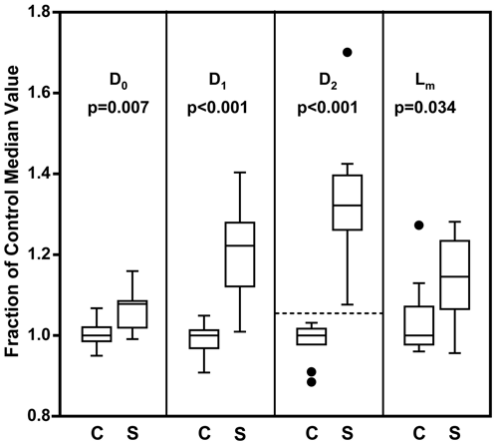
Boxplots of histological data from the 10 control (C) and 10 smoke-exposed (S) mice. D_0_ is the mean equivalent diameter, D_1_ and D_2_ are weighted indexes of airspace size distribution, and L_m_ is the mean linear intercept. Data were normalized to the median values of the control group. The p-values indicate the significance of the ability of each method to discern between the treatment groups. The dotted line was added to illustrate the lack of overlap between groups in the D_2_ results. In these boxplots, the box vertical dimensions represent the first and third quartiles, the line inside the box represents the median (second quartile), the bars represent the largest and smallest non-outliers (within 1.5 times the interquartile range), and the points represent outliers (beyond 1.5 times the interquartile range).

Boxplots of the results are shown in Figure 6, comparing the control (C) and smoking (S) groups with accompanying ANOVA p-values. The difference between the control and smoking groups is statistically significant in L_m_ (p-value = 0.034) but with a clear overlap in individual values between the two groups. By comparison, the D_2_ results showed a higher degree of significance from an ANOVA model (p-value<0.001) with a clear separation between the control and smoking groups (as emphasized by the horizontal dashed line). This was verified by a significance of interaction test from an ANOVA model, which indicated that the separation of treatment groups was significantly more pronounced for D_2_ than it was for L_m_ (p-value<0.001). ANOVA tests determined that D_0_ and D_1_ were also better at distinguishing treatment groups than L_m_ but were not better than D_2_. In addition, the sensitivity and specificity results from the linear discriminate analysis showed that the sensitivity of D_2_ exceeded that of L_m_ (80% vs. 60%) as did the specificity (100% vs. 80%), indicating that D_2_ was better at predicting which mice belonged to the control vs. smoke-exposed group.

## Discussion

In this study we compared two methods for quantifying airspace enlargement in smoke-exposed mice. We followed standard procedures for lung tissue sample preparation, image acquisition, and L_m_ analysis. Following this, we calculated the new index, D_2_, on the same images to compare how well the two methods separate the smoke-exposed and control groups. Our results show that D_2_ was better able to distinguish between the groups (see Figure 6), and this is attributed to the fact that D_2_ is weighted by enlarged airspaces and is therefore a reflection of the airspace size distribution. L_m_, on the other hand, is a measure of the intra-alveolar septal wall mean free path and tends to mask the presence of sparse, enlarged airspaces. We emphasize that D_2_ does not provide information about the actual airspace geometries; rather, it simply offers a more sensitive metric of airspace enlargement.

A manual validation of the automated D_2_ measurements was performed to assure that the automation did not misinterpret features and would not adversely affect the results. Figures 4 and 5, with accompanying statistical analysis, confirm that full automation did not introduce appreciable errors. We note that the difference in scatter in the top panel of Figure 5 (the D_0_ comparison) versus that of the bottom panel (the D_2_ comparison) illustrates that small discrepancies in thresholding, particularly of the smallest airspaces (cf. Figures 4B and 4C), are outweighed by the effects of the largest airspaces and are, therefore, generally not significant. This point underscores the robustness of the automated method. Still, there may be cases when a semi-automated implementation may be necessary, such as situations of poor image quality or images that include large blood vessels or conducting airways. We note that the image processing method employed herein differs somewhat from that originally used in Ref. [18]. There, the authors applied a watershed segmentation to the lung histology images to define the airspace boundaries. Although easy to automate, this type of segmentation may not realistically represent the airway architecture. For example, airway walls were represented as thin lines while the tissue itself was either incorporated into the airways or was segmented into additional “airspaces.” Another problem is that this segmentation does not allow for “free ends” which are generally alveolar openings from alveolar ducts [6]; rather, it connects the free ends, resulting in artificial subdivision of airspaces. Herein, we implemented and automated the method of simple thresholding to more faithfully define the tissue boundaries as depicted in the histology images [31].

The full automation of D_2_ calculations has eliminated intermediate, time-intensive steps, such as point counting, without sacrificing accuracy. This has two primary advantages over manual or semi-automated methods. 1) Full automation eliminates the potential for operator bias by removing the opportunity to make decisions that might skew the results. The only prospects for bias would be in the tissue sampling or acquisition of the images themselves, which can be avoided through strict implementation of random and blinded means. 2) Full automation is much faster and is relatively simple to employ using existing technology and computational methods. In this study, calculation of D_2_ starting from the raw images was at least 24x faster than the semi-automated L_m_ measurements, as D_2_ required approximately 5 seconds per image and L_m_ required 2–3 minutes per image.

Both L_m_ and D_2_ have strengths that can be exploited in studies of lung structure. L_m_ has the advantage of providing a quantitative measure of the volume to surface-area ratio. D_2_, on the other hand, has advantages of sensitivity, reliability, and speed when measuring airspace enlargement. With rigorous D_2_ validation studies such as presented herein, we anticipate that D_2_ and L_m_ can be used in tandem as quantitative measures in emphysema assessment to provide high sensitivity to disease state, as well as quantitative information about average airspace dimensions, respectively. By further probing the sensitivity limitations of D_2_, a useful lower bound of its practical implementation can be determined. Therefore, future work should investigate the limits of D_2_ sensitivity in, for example, disease states of minimal severity. The ability to detect very early stages of airway enlargement may provide additional biomarker candidates associated with disease onset and progression.
